# Pharmacokinetics of the SABRE agent 4,6-d_2_-nicotinamide and also nicotinamide in rats following oral and intravenous administration

**DOI:** 10.1016/j.ejps.2019.05.004

**Published:** 2019-07-01

**Authors:** Inna V. Linnik, Peter J. Rayner, Ruth A. Stow, Simon B. Duckett, Graham M.T. Cheetham

**Affiliations:** a Centre for Hyperpolarisation in Magnetic Resonance (CHyM), Department of Chemistry, University of York, Heslington YO10 5DD, UK; b Covance Laboratories, Harrogate, North Yorkshire HG3 1PY, UK

**Keywords:** d_2_-NA, 4,6-d_2_-nicotinamide, NA, protio-nicotinamide, SABRE, Signal Amplification By Reversible Exchange, MRI, Magnetic Resonance Imaging, IV, intravenous, IP, intraperitoneal, PO, *per os* (oral), LLOQ, Lower Limit of Quantification, ULOQ, Upper Limit of Quantification, SABRE, Hyperpolarization, Deuteration, Pharmacokinetics, Nicotinamide

## Abstract

To prepare the way for using the isotopically labelled SABRE hyperpolarized 4,6-d_2_-nicotinamide as an MRI agent in humans we have performed an *in-vivo* study to measure its pharmacokinetics in the plasma of healthy rats after intravenous and oral administration. Male Han Wistar rats were dosed with either 4,6-d_2_-nicotinamide or the corresponding control, non-labelled nicotinamide, and plasma samples were obtained at eight time points for up to 24 h after administration. Pharmacokinetic parameters were determined from agent concentration-*versus*-time data for both 4,6-d_2_-nicotinamide and nicotinamide. 4,6-d_2_-Nicotinamide proved to be well tolerated regardless of route of administration at the concentrations used (20, 80 and 120 mg/kg). Pharmacokinetic parameters were similar after oral and intravenous administration and similar to those obtained for nicotinamide. Analysis of nicotinamide plasma concentrations after dosing 4,6-d_2_-nicotinamide intravenously demonstrates a reversible exchange of endogenous nicotinamide by this labelled agent over the time-course of our assays. Supported by a large body of evidence for the safety of nicotinamide when dosed orally in humans, we conclude that 4,6-d_2_-nicotinamide can also be safely administered intravenously, which will provide significant benefit when using this agent for planned imaging studies in humans.

## Graphical abstract

Mean concentrations of (a) 4,6-d2-nicotinamide and (b) nicotinamide in the plasma of male rats following a single IV administration of 4,6-d2-nicotinamide at nominal dose levels of 20 mg/kg (), 60 mg/kg () and 120 mg/kg (). Endogenous NA is released after intravenous administration of d2-NA.

**Figure:**
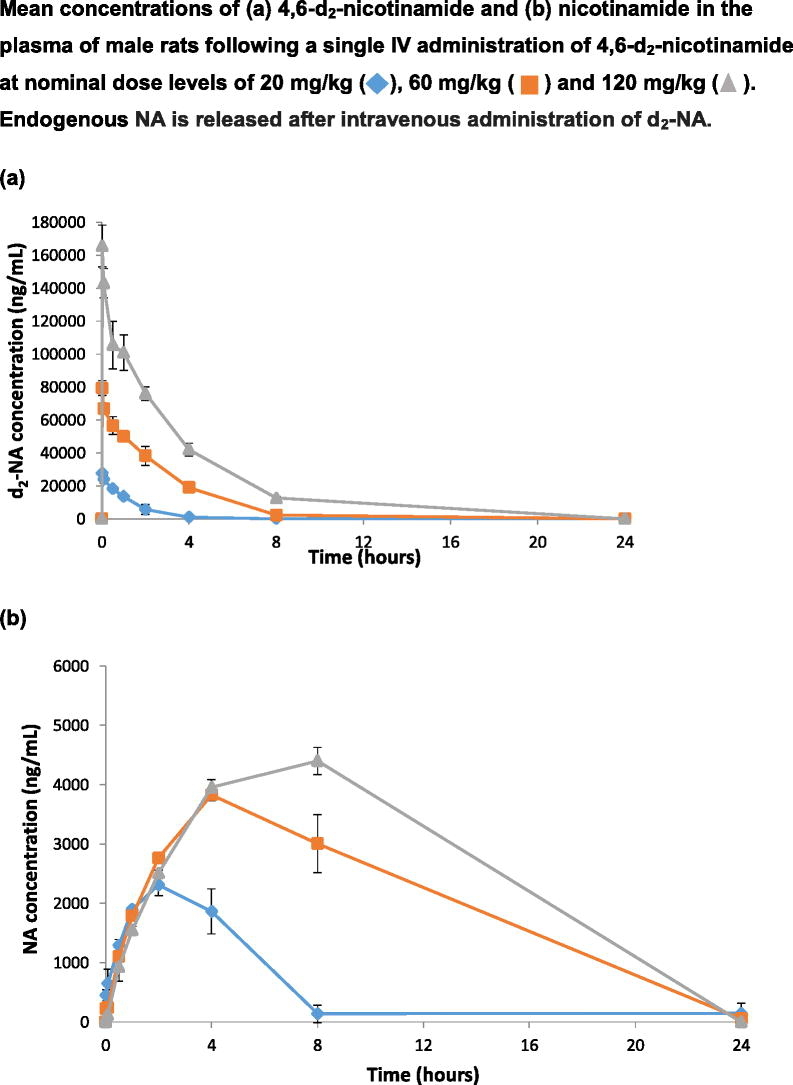

## 1 Introduction

Over the last few decades Magnetic Resonance Imaging (MRI) has evolved to become an extremely important technique that allows researchers to obtain anatomical, functional and metabolic information. However, even after this tremendous success, its applicability is limited due to low sensitivity and high cost. The low sensitivity is derived from the fact that nuclei possess little intrinsic magnetization and interact weakly with a magnetic field. Dynamic nuclear polarization (DNP) is now an established technique for dramatically increasing the sensitivity of Magnetic Resonance Imaging and spectroscopy for the study of *in-vivo* metabolism (Kurhanewicz et al., 2011; Gallagher et al., 2011). Using pyruvate as a hyperpolarization agent, DNP has demonstrated that detailed clinical MRI images can be obtained in humans (Grist et al., 2019; Daniels et al., 2016). We have sought to overcome the low sensitivity problem by developing similarly highly visible agents through a method of hyperpolarization called SABRE (Adams et al., 2009). SABRE's rapidity and potentially low cast mean it too has the potential to revolutionise clinical MRI, and related MR methods by improving the strength of the detected response by over four orders of magnitude from its normal level as found on a routine 1.5 T clinical MRI system (Rayner et al., 2017; Duckett and Mewis, 2013). SABRE reflects a novel approach that does not change the chemical identity of the agent it hyperpolarizes but in stead equilibrates polarization between *p*-H_2_ and the selected agent through binding to an iridium centre (Adams et al., 2009) in a novel form of catalysis. With this sensitivity gain, SABRE hyperpolarization of ^1^H, ^13^C, ^15^N, ^31^P atoms (Iali et al., 2018; Roy et al., 2018; Olaru et al., 2017) in labelled drugs or agents is expected to radically enhance their future detection by MRI and allow imaging of metabolic and physiologic processes of substrates *in vivo*. It may also enable *in vivo* nano-chemistry and metabolomics, and decreases the time needed to observe a metabolite concentration change (Viale and Aime, 2010; Kurhanewicz et al., 2011).

We have chemically developed nicotinamide (NA) using NMR assessment as a SABRE agent for future MRI applications. NA belongs to the niacin family of compounds and is a common nutrient. It is also described as belonging to a class of compounds called histone deacetylase (HDAC) inhibitors, which have been shown to protect the central nervous system in rodent models of Parkinson's and Huntington's diseases (Didonna and Opal, 2014). Clinical trials are currently underway to learn whether HDAC inhibitors help ALS and Huntington's patients (Wild and Tabriziz, 2014). Our SABRE agent, 4,6-d_2_-nicotinamide (d_2_-NA), has the same chemical structure as NA except that two protons at positions 4 and 6 on the aromatic ring have been replaced by deuterium (Rayner et al., 2017). Though numerous publications have discussed the advantages and disadvantages of deuterated drugs (Liu et al., 2017; Gant, 2014; Katsnelson, 2013; Sanderson, 2009; Foster, 1985) and several companies focus on the use of deuterated drugs for various conditions, we use deuteration solely to enhance the SABRE hyperpolarization properties of NA. The deuterium positions are magnetically silent and serve to focus magnetization on the two remaining aromatic protons. The ability of SABRE to hyperpolarize protons, as in d_2_-NA, makes it applicable as a method to make agents for use in all hospital MRI scanners. One of the benefits of deuterium labelling is that the magnetic longitudinal relaxation time of d_2_-NA is considerably extended when compared to NA (Rayner et al., 2017).

One factor that was important in selecting NA is that it is an intrinsically safe clinical agent that has already been used in pharmacological doses over many years with a low incidence of side effects and toxicity (Final Report of the Safety Assessment of Niacinamide and Niacin, 2005). Importantly, previous literature reports provide considerable evidence that NA reflects a safe therapy to use when given at adult doses of up to 10 g/day orally (Knip et al., 2000) and is commonly used in clinical trials at oral (PO) doses of ≥3 g/day in humans for various indications. Doses (up to 6 g/day) have been used in combination with radiotherapy (Dragovic et al., 1995). The pharmacokinetics of NA are known to be dependent on dose, species, sex and route of administration. Safe doses in rats have previously been characterized: 750 mg/kg for subcutaneous injection and 1000 mg/kg for IP injection (Ayouba et al., 1999) Furthermore, intravenous (IV) doses of 750 mg/kg in rat models of stroke (Sakakibara et al., 2000) have been used. An *in vitro* cytotoxicity study demonstrated that it is possible to create a biocompatible SABRE bolus of d_2_-NA and that deuteration of NA does not increase the toxicity compared to the NA (Manoharan et al., 2018).

We are now seeking to progress d_2_-NA to first-in-human exploratory MR imaging studies, where it is anticipated that this agent will be used intravenously as a bolus injection to observe perfusion in the heart or brain, for example. In order to pave the way for using d_2_-NA we intend to conduct two *in-vivo* preclinical studies: a PK study and a single dose toxicity study in rats. At sub-therapeutic doses, guided by a body of clinical data for NA in humans and current regulatory guidelines, only rodent data may be required to support the use of d_2_-NA in humans. We report here the results of the first of these whereby the pharmacokinetics of d_2_-NA in the plasma of healthy rats after PO and IV administration was measured. Data describing IV administration of NA in human is limited and there is no corresponding information known for d_2_-NA. The pharmacokinetics study of d_2_-NA is required to predict its efficacy and the duration of its effects, especially in comparison to NA, and to provide supportive data on the mechanisms of drug action, to predict side-effects and for the extrapolation of results from laboratory animals to man. In this study, we used sub-therapeutic doses between 20 and 120 mg/kg in rats. The main objective is to measure plasma concentrations of d_2_-NA following IV and PO administration and to compare these to the pharmacokinetics of NA.

## 2 Materials and methods

This is a non-regulatory study for which a claim of GLP compliance is not been made. However, the laboratory procedures used were consistent with International Standards of GLP.

### 2.1 Animals

Fifteen male Han Wistar rats were obtained from Charles River Laboratories, Margate, UK. Animals were all in the target weight range of 163 to 215 g at dosing. Diet was removed at the end of the working day prior to each dose occasion, and returned 4 h after dosing.

### 2.2 Agents used

Both d_2_-NA and NA were formulated by dissolving appropriate amounts of each in 0.9% physiological saline and were assessed visually for complete dissolution. No cloudy suspension or particles were visible (Table 1).

**Table 1:** Agents used for this PK study.

Test substance name	4,6-d_2_-Nicotinamide	Nicotinamide
Acronym	d_2_-NA	NA
Chemical form	Free compound	Free compound
Physical form	Solid	Solid
Chemical structure		
Molecular formula	C_6_H_4_D_2_N_2_O	C_6_H_6_N_2_O
Molecular weight	124.1 g/mol	122.1 g/mol
Chemical purity	99%	99%

### 2.3 Experimental design

Each animal received a single administration (Table 2). PO doses were administered by gavage at a nominal dose volume of 5 ml/kg. IV doses were administered *via* a lateral tail vein, at a nominal dose volume of 2 ml/kg, as a bolus injection. Body weights were recorded the day after arrival and before dose administration and used for calculation of doses. Samples of blood (nominally 150 μl) were collected from the jugular vein of each animal. For PO dosing this was done at 0.25, 0.5, 1, 2, 4, 6, 8 and 24 h post dose. For IV dosing this was at 0.016, 0.083, 0.5, 1, 2, 4, 8 and 24 h post dose. The actual blood collection time was used in the subsequent PK data analysis. Blood was collected into tubes containing the K_2_EDTA anticoagulant and stored at room temperature. Blood was centrifuged (1500*g*, 10 min, 4 °C) to produce plasma for analysis and residual blood cells were discarded.

**Table 2:** Dose groups for d_2_-NA and NA.

Dose group	Agent used	Dose route	Dose level	Number of animals
			mg/kg	Males
A	NA	IV	120	3
B	d_2_-NA	IV	120	3
C	d_2_-NA	PO	120	3
D	d_2_-NA	IV	60	3
E	d_2_-NA	IV	20	3

### 2.4 Blood plasma assay for d_2_-NA and NA

Initial NA concentration quantification revealed an endogenous NA background peak during calibration, the intensity of which is consistent with a level of approximately 50 ng/ml. Concentrations of d_2_-NA and NA were subsequently determined by the now qualified LC-MS/MS method of Table 3 which involved the following steps:

1. Transfer of a 10 μl aliquot into a clean 2 ml 9-well collection plate
2. Addition of 25 μl I-B (500 ng/ml). Addition of 25 μl methanol to all blanks
3. Vortex mix plate (*ca.* 1 min)
4. Addition of 80 μl methanol to all wells
5. Vortex mix plate (*ca.* 2 min)
6. Centrifuge 2000*g* plate for 10 min at room temperature
7. Transfer 40 μl of supernatant to a clean 1.2 ml 96-well collection plate
8. Dilute with 160 μl 10 mM of ammonium acetate:ammonium hydroxide (100:0.5 v/v) solution
9. Vortex mix plate (*ca.* 2 min)
10. Complete analysis by LC/MS-MS.

**Table 3:** Mass spectrometer setup.

Mass spectrometer	Sciex 6500
Ionisation interface and temperature	TISP 650 °C
LC system	Shimadzu Nexera
Mobile phase A	10 mM ammonium acetate: ammonium hydroxide (100:0.5)
Mobile phase B	Methanol
Weak wash	Methanol:water (50:50)
Strong wash	2-Propanol:methanol:THF:water (25:25:25:25 v/v/v/v)
Auxiliary wash	Methanol:water (50:50)
Injection volume	20 μl
Analytical column	Phenomenex Gemini 3 μm NX-C18, 50 × 2 mm
Column temperature	40 °C
Flow rate	0.6 ml/min
Sample temperature	5 °C


Gradient profile	Time (min)	% aqueous phase
	0	95
	0.2	95
	2	2
	2.5	2
	2.6	95
	3.2	95

### 2.5 Pharmacokinetic analysis

Pharmacokinetic analysis of concentrations of d_2_-NA and NA in blood and plasma was performed using the validated non-compartmental pharmacokinetic analysis program Phoenix WinNonLin, version 6.4 (Certara, St Louis, Missouri, USA).

## 3 Results

No clinical signs were observed to be associated with the IV or PO administration of d_2_-NA or NA at doses up to 120 mg/kg (Table 2) to fifteen animals. A complete list of the associated pharmacokinetic parameters determined for each of these dose groups are presented in Table 4. For all dose groups, the mean volume of distribution was high compared to the blood volume in the rat (*ca* 54 ml/kg (Davies and Morris, 1993)), suggesting that test compound was distributed into body water or tissues. The mean plasma clearance of all groups was lower than liver blood flow in the rat (*ca* 3000 ml/h/kg (Davies and Morris, 1993)) suggesting passive clearance from the systemic circulation and some distribution into body tissues and/or saturation of clearance mechanisms. The following results were calculated for each group.

**Table 4:** Pharmacokinetic parameters.

Dose group	Cmax	t_½_	AUC_(0–t)_	AUC_(0–∞)_	C_0_	V_z_	Cl
	ng/ml	h	h·ng/ml	h·ng/ml	ng/ml	mL/kg	mL/h/kg
A	168,000	4.06	573,000	774,000	178,000	911	163
B	166,000	2.33	416,000	459,000	172,000	877	262
C	96,000	2.00	418,000	386,000	–	–	–
D	79,400	2.23	196,000	197,000	82,700	963	307
E	27,700	0.83	34,000	34,800	28,700	691	586

C_max_: maximum plasma concentration observed.

t_½_: the terminal elimination phase half-life was determined by linear regression of at least three data points (not including C_max_) the terminal portion of the log-linear concentration *vs* time curve.

AUC_(0–t)_: area under the concentration-time curve calculated from 0 to t, where t is the time of the last measurable concentration, was calculated by non-compartmental analysis using the log/linear trapezoidal rule.

AUC_(0–∞)_: area under the concentration-time curve extrapolated to infinite time was calculated by non-compartmental analysis using the log/linear trapezoidal rule.

C_0_: plasma concentration at time zero following the IV dose was obtained by back extrapolation of the first two plasma concentrations.

V_z_: volume of distribution based on the terminal phase following IV dosing, calculated using the equation: $V_{z}=\frac{\mathrm{Dose}}{\lambda_{z}\times {\mathrm{AUC}}_{\left(0-\infty \right)}}$.

Cl: total body clearance following IV dosing was calculated using the equation: $\mathrm{Cl}=\frac{\mathrm{Dose}}{{\mathrm{AUC}}_{\left(0-\infty \right)}}$.

### 3.1 Control group A (120 mg/kg NA, IV)

**Fig. 1:**
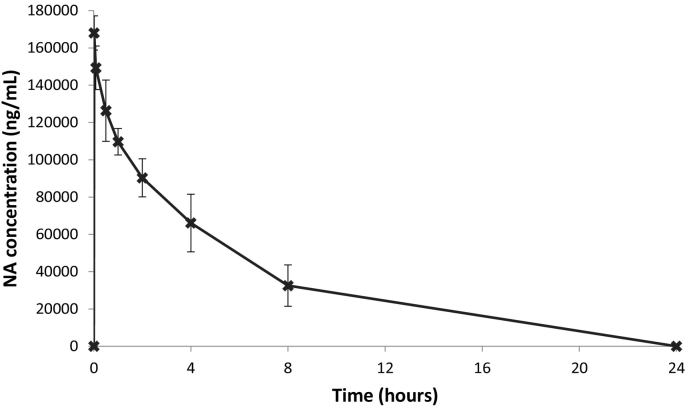
Mean concentrations of NA in the plasma of male rats (n = 3) following a single IV dose at a nominal dose level of 120 mg/kg (group A).

Although used as a control group in this study, not much is known about the pharmacokinetics of NA after IV dosing. Almost all preclinical and clinical *in-vivo* data have resulted from PO dosing regimes. We report here that a mean plasma concentration of NA of 168,000 ± 9170 ng/ml is observed 0.016 h post-dose, which suggests a mean plasma C_0_ of 178,000 ± 16,700 ng/ml. NA was still detected in the plasma of all three animals 8 h post-dose (32,500 ± 1100 ng/ml) but was below the LLOQ of 50 ng/ml by 24 h post-dose. These data are presented in Table 4 and Fig. 1. In comparison with group B, exposure to NA was 1.7 times higher than exposure to d_2_-NA after dosing of d_2_-NA at the same dose level of 120 mg/kg as judged by AUC data, even though Cmax data are consistent (Table 4.). This difference is driven by a significantly longer half-life observed for NA (4.0 h for Group A *versus* 2.3 h for Group B). Our interpretation of this data is that d_2_-NA may be metabolised and cleared more quickly from plasma than NA.

### 3.2 Group B (120 mg/kg d_2_-NA, IV)

After an IV administration of d_2_-NA at a nominal dose level of 120 mg/kg, a mean plasma concentration of d_2_-NA of 166,000 ± 12,700 ng/ml was observed 0.016 h post-dose, which is approximately 2-times higher than for group D where the dose is (60 mg/ml) and 6-times higher than for group E (20 mg/ml). This indicates dose response proportionality. A mean plasma C_0_ level of 172,000 ± 16,700 ng/ml was detected which compares well with the C_0_ of 178,000 ± 16,700 ng/ml of control group A (120 mg/kg NA, IV). Increased d_2_-NA levels were still detected in all three animals at 8 h post-dose but the value falls to below 50 ng/ml in all three animals by 24 h post-dose.

Interestingly, although only d_2_-NA was dosed in this group, low levels of NA were detected in two out of the three animals at the first sampling point (mean 73 ± 63.2 ng/ml), increasing to a mean C_max_ of 4400 ± 229 ng/ml (*ca* 2.7% of d_2_-NA) at 8 h post-dose (Fig. 3). An estimate of the t_½_ and corresponding AUC_(0–∞)_ for NA could not be determined from a simple analysis of the NA pharmacokinetic profile.

### 3.3 Group C (120 mg/kg, d_2_-NA PO)

Following PO administration of d_2_-NA at a nominal dose level of 120 mg/kg, the plasma of all animals demonstrated exposure to the agent. A mean maximum (C_max_) plasma concentration of 96,000 ± 31,500 ng/ml was reached within 0.5 to 4 h (median of 0.5 h). By 24 h post dose, the mean plasma concentrations of d_2_-NA had declined to a baseline value of 21.7 ± 37.6 ng/ml, 0.02% of C_max_. Hence the mean terminal elimination half-life is 2.00 h suggesting rapid elimination, which is comparable to that found for group B, after an IV dose at the same initial level. The mean AUC_(0–t)_ was 20,100 ± 1190 h·ng/ml, is also comparable to the systemic exposure observed after a 120 mg/kg IV dose (group B).

### 3.4 Group D (60 mg/kg d_2_-NA, IV)

After an IV administration of d_2_-NA at a nominal dose level of 60 mg/kg, a mean plasma concentration of d_2_-NA of 79,400 ± 4540 ng/ml was observed at (C_t_), 0.016 h post-dose. d_2_-NA was still detected in all three animals at 8 h post-dose but had been eliminated in two out of three by 24 h post-dose. The terminal half-life (t_½_) was 2.23 ± 0.78 h. As for group B, NA was detected in all three animals at the first sampling point, albeit at very low concentrations: mean 225 ± 40.9 ng/ml. However, the concentration of NA increased to a mean of 3830 ± 97.1 ng/ml (*ca* 4.8% of d_2_-NA) at 4 h post-dose.

### 3.5 Group E (20 mg/kg d_2_-NA, IV)

Following IV administration of d_2_-NA at a nominal dose level of 20 mg/kg, a mean plasma concentration of d_2_-NA of 27,700 ng/ml, was observed at the first sampling point (C_t_), 0.016 h (1 min) post-dose. When extrapolated back to time zero (C_0_), this corresponds to a mean plasma concentration of 28,700 ± 657 ng/ml. d_2_-NA again proved to be eliminated in all animals by 24 h post-dose. The mean areas under the plasma concentration time curves, AUC_(0–t)_ and AUC_(0–∞)_ were 34,000 ± 5370 h·ng/ml and 34,800 ± 6460 h·ng/ml, respectively, with a corresponding terminal half-life (t_½_) of 0.828 ± 0.175 h. As now expected, NA was detected in all three animals at the first sampling point and a mean maximum concentration of 2310 ± 180 ng/ml (*ca* 8% of parent compound) was achieved at *ca* 2 h post-dose. These data did not lend themselves to t½ or AUC_(0–∞)_ determination.

## 4 Discussion

**Fig. 2:**
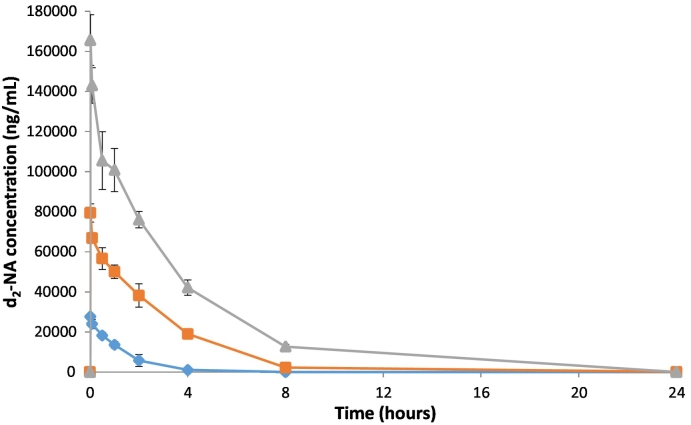
Mean concentrations of d_2_-NA in the plasma of male rats (n = 3) following a single IV administration of d_2_-NA at nominal dose levels of 20 mg/kg (group E ), 60 mg/kg (group D ) and 120 mg/kg (group B ).

The dose normalised AUC_(0–∞)_ data suggests that at 60 and 120 mg/kg levels the exposure to d_2_-NA is proportional to the dose administered (Fig. 2). The sub-proportional exposure, in terms of AUC_(0–∞)_, at the lowest dose level, 20 mg/kg, is possibly due to concentrations being smaller relative to the animals NA pool, but we note they are also close to the limits of quantification, and hence there were fewer detectable data points; the corresponding pharmacokinetic parameters are therefore less well defined. The measured exposure level after a PO dose of d_2_-NA was however directly comparable to that of IV exposure at 120 mg/kg and this is reflected in the measured bioavailability of 98.7%. Hence, the IV and PO routes should result in similar agent response behaviour. In fact, C_max_ concentrations of NA after IV administration were comparable to those of d_2_-NA after d_2_-NA administration.

NA occurs as a component of a variety of biological systems and as a part of the coenzyme nicotinamide adenine dinucleotide (NADH/NAD^+^): it is crucial to life (Belenky et al., 2007). Once ingested, nicotinamide undergoes a series of reactions that transform it into NAD, which can then undergo a transformation to form NADP^+^. However, rats and humans can produce NAD^+^ from the amino acid tryptophan and niacin without ingestion of nicotinamide (Williams et al., 2015). As part of our analysis, we estimated the endogenous level of NA to be approximately 50 ng/ml in rat plasma and set the Lower Limit of Quantification (LLOQ) for our analysis at this level. The ULOQ was set at 250,000 ng/ml.

**Fig. 3:**
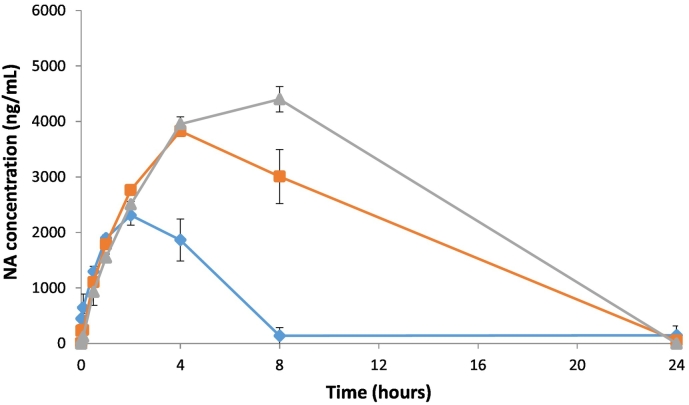
Mean concentrations of NA in the plasma of male rats (n = 3) following a single IV administration of d_2_-NA at nominal dose levels of 20 mg/kg (group E ), 60 mg/kg (group D ) and 120 mg/kg (group B ). Endogenous NA is released after intravenous administration of d_2_-NA.

This study aims to determine the pharmacokinetics of d_2_-NA after dosing *in-vivo* in comparison to a control group of NA. However, one of the most interesting findings is that dosing of d_2_-NA elicits a release or production of NA *in-vivo*, isolated in blood plasma of rats from groups B, D and E (Fig. 3). A significant level of NA (C_max_ 2310 ± 180 ng/ml) was observed 2 h after dosing d_2_-NA at 20 mg/kg (group E). This is approximately 8% of the level of d_2_-NA dosed. This NA concentration reduced to zero after 8 h and did not fit a normal pharmacokinetic profile. For the 60 mg/kg d_2_-NA group (D) the level of NA observed rose to 3830 ± 97.1 ng/ml (4.8% of d_2_-NA) 4 h post-dose while for a 120 mg/kg dose of d_2_-NA (group B) a mean C_max_ of 4400 ± 229 ng/ml (2.7% of d_2_-NA) was found at 8 h post-dose (Fig. 3).

Our interpretation of these data is that at all dose levels used in this study d_2_-NA exchanges and partially replaces the “pool” of endogenous NA in body tissues, resulting in a release of NA into the plasma during the time course of our assay. The data presented in Fig. 3 shows how the concentrations of NA, which are significantly in excess of the estimated endogenous NA levels and LLOQ of 50 ng/ml, vary with time after dosing with d_2_-NA. Endogenous NA, which is replaced by d_2_-NA and released to the plasma, is linked to the response level in terms of C_max_ to the levels of d_2_-NA injected. In comparison to the concentration-*versus*-time plasma levels of d_2_-NA (Fig. 2) there is a delay of 2–8 h to reach C_max_ for the released endogenous NA (Fig. 3) which suggest that exchange with the endogenous pool is slow. The shapes of these NA concentration-*versus*-time profiles are consistent with this slow replacement of NA in the endogenous pool by d_2_-NA followed by simultaneous clearance of the excess released endogenous NA. Importantly, the plasma levels of NA after dosing d_2_-NA return to baseline by 24-h, indicating the clearance of all dosed d_2_-NA and the released endogenous NA.

The presence of NA when d_2_-NA is dosed in rats is not a result of contamination of the dose samples or loss of the deuterium label from d_2_-NA. In a bioanalytical study we have demonstrated that the deuterium label of d_2_-NA is stable in rat blood plasma for significantly longer than 24 h (data not shown).

## 5 Conclusions

**Fig. 4:**
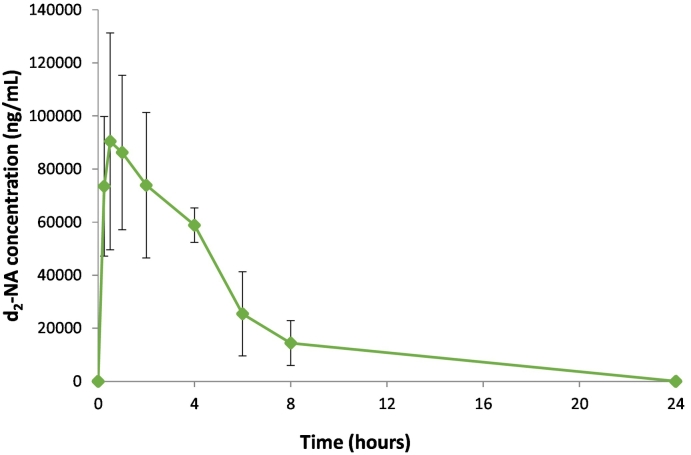
Mean concentrations of d_2_-NA in the plasma of male rats (n = 3) following a single PO administration at a nominal dose level of 120 mg/kg (group C).

Importantly, this study has demonstrated that d_2_-NA is well tolerated regardless of route of administration at the concentrations used (20, 80 and 120 mg/kg bodyweight) and that pharmacokinetic parameters of d_2_-NA are similar after PO (Fig. 4) and IV administration and similar to those obtained for NA after IV dosing. Taken together the pharmacokinetic data suggest that there are no significant differences between dosing either NA or d_2_-NA. This is important as it helps us to bridge the large body of known clinical data for the use of NA *via* PO dosing with our intended IV administration of hyperpolarized d_2_-NA as an MRI imaging agent.

Concerning the significant presence of NA after dosing of d_2_-NA our conclusion is that an endogenous pool of NA is partially replaced by d_2_-NA in a dose dependent fashion (Fig. 3). We have also shown that this process is reversible on the timescale of our study after a single dose at time zero and hypothesize that newly synthesized endogenous NA refills the endogenous cellular or body tissue pool. Both d_2_-NA and the released endogenous NA are completely cleared within 24 h after IV dosing and a normal equilibrium is restored to the pool of endogenous NA.

This pharmacokinetic study is the first of two *in-vivo* rat studies we will be conducting. It will be followed by a single-dose IV bolus study of d_2_-NA in rats followed by a 14-day recovery period, necropsy and histopathology, to determine the safety toxicity profile of this d_2_-NA after hyperpolarization and extraction from the SABRE catalyst reaction mix. We will use a biphasic separation procedure to do this (modified from Manoharan et al., 2019). This toxicity study will be a pivotal preclinical study to determine if this d_2_-NA agent can be progressed clinically in humans as an MRI imaging agent of well perfused organs or tissues such as the heart, brain or kidney.
